# A Model of Exposure to Extreme Environmental Heat Uncovers the Human Transcriptome to Heat Stress

**DOI:** 10.1038/s41598-017-09819-5

**Published:** 2017-08-25

**Authors:** Abderrezak Bouchama, Mohammad Azhar Aziz, Saeed Al Mahri, Musa Nur Gabere, Meshan Al Dlamy, Sameer Mohammad, Mashael Al Abbad, Mohamed Hussein

**Affiliations:** 1 King Abdullah International Medical Research Center/King Saud bin Abdulaziz University for Health Sciences, Experimental Medicine Department-MNGHA, Riyadh, 11426 Saudi Arabia; 2 King Abdullah International Medical Research Center/King Saud bin Abdulaziz University for Health Sciences, Colorectal Cancer Research Program-MNGHA, Riyadh, 11426 Saudi Arabia; 3 King Abdullah International Medical Research Center/King Saud bin Abdulaziz University for Health Sciences, Biostatistics and Bioinformatics Department-MNGHA, Riyadh, 11426 Saudi Arabia; 4 King Abdullah International Medical Research Center/King Saud bin Abdulaziz University for Health Sciences, Research Trauma Project-MNGHA, Riyadh, 11426 Saudi Arabia

**Keywords:** Gene expression, Homeostasis

## Abstract

The molecular mechanisms by which individuals subjected to environmental heat stress either recover or develop heat-related complications are not well understood. We analysed the changes in blood mononuclear gene expression patterns in human volunteers exposed to extreme heat in a sauna (temperature of 75.7 ± 0.86 °C). Our analysis reveals that expression changes occur rapidly with no significant increase in core temperature and continue to amplify one hour after the end of heat stress. The reprogramed transcriptome was predominantly inhibitory, as more than two-thirds of the expressed genes were suppressed. The differentially expressed genes encoded proteins that function in stress-associated pathways; including proteostasis, energy metabolism, cell growth and proliferation, and cell death, and survival. The transcriptome also included mitochondrial dysfunction, altered protein synthesis, and reduced expression of genes -related to immune function. The findings reveal the human transcriptomic response to heat and highlight changes that might underlie the health outcomes observed during heat waves.

## Introduction

Environmental temperatures are increasing throughout the world, including in temperate climatic zones, raising concerns about how increasing temperatures might affect human health, given the associated health risks^1, 2^. Indeed, exposure to high ambient temperatures can result in high morbidity and mortality^2–5^. In July 1995, a heat wave in the USA caused 700 excess deaths and more than 3000 emergency room visits in the city of Chicago^5^. More recently, in 2003 and 2010, respectively, two heat wave-related disasters affected Western Europe and Russia, resulting in 70,000 and 55,000 excess deaths^2, 3^. Analysis of “excess” deaths during heat waves revealed that heatstroke, a condition characterized by rapidly increasing body temperature and multiple organ failure, alone accounted for one-third of the fatalities, whereas the remainder were attributed to heat-aggravated medical conditions, particularly cardiovascular and pulmonary diseases^4, 5^. Despite this established relationship between high environmental temperature and morbidity and mortality, the mechanisms by which heat contributes to clinical outcome are not fully understood^6, 7^. It is neither easy to perform studies in humans with heat injury, nor to interpret the results, because the dose and duration of heat exposure, as well as the precise onset of heat-related complications are difficult to determine. In addition, the data are often confounded by comorbid illnesses and concurrent medications^4–7^. Hence, as yet, there are no specific targeted preventive and/or therapeutic measures available other than avoiding heat exposure and physical cooling^4, 5, 7^.

Heat stress triggers a range of adaptive physiological and cellular mechanisms, including thermoregulation and the cellular stress response (CSR), particularly the induction of heat shock proteins (HSPs) to prevent hyperthermia, cellular damage and death^7–10^. It is currently believed that cardiovascular stress, imposed by thermoregulation, underlies the maladaptive response to heat stress – a marked increase in cardiac output is needed to accelerate the transport of heat to the skin, and thereby to the environment, and this may progress to cardiovascular failure and death^7, 9^. At the cellular level, cytotoxicity caused by heat, failure to increase the expression of HSPs, excessive inflammation, and the activation of coagulation have also been implicated in the pathogenesis of heat injury; including its associated tissue damage, and death^7^. However, the human stress response to passive exposure to environmental heat, by which heat-stressed individuals recover or develop fatal heat-related complications, has not been characterized at the transcriptomic level; such an analysis might help us to understand the molecular mechanisms that underlie the pathogenesis of heat-related morbidity and mortality.

Transcriptomics have emerged as a powerful approach for investigating the molecular response to environmental stressors, including heat^11–14^. However, most genomic studies of the CSR to heat stress have used model organisms, such as flies, worms and yeast, or used isolated human cells grown in culture^10, 12, 14–20^. These studies revealed that cells activate ancient cellular and molecular protective mechanisms that have evolved from prokaryotes to mammalian cells; resulting in rapid and transient reprioritization of the gene expression program in response to stress. Genes involved in growth-related processes are suppressed. Energy resources are redirected to stress-related functions to allow cells to survive the changing environment. A general stress response common to most cells was characterized and found to include the identification and repair of misfolded or aggregated proteins or their transport to sites of degradation, cell cycle control to allow stabilization and/or repair of altered DNA and chromatin, and regulation of energy metabolism and redox state of the cells^12–14^. Moreover, in complex multicellular organisms, the CSR can activate a cell death program, if the cellular effects of the stress cannot be mitigated^10, 18–20^.

Human studies have focused on exertional heat stress in athletes, and have generally been limited to specific pathways, such as the HSP and/or the inflammatory response^21–24^. Further, the findings were often confounded with muscle injury associated with vigorous exercise^22, 23^. Nonetheless, several components of the general stress response were identified that vary with the methods of exertion and the arrays utilized. These include the expression of genes involved in HSPs, innate immunity and mitochondrial function; as well as genes related to growth and tissue repair^21–25^.

Using a combination of a unique human model of heat-stress with a full recovery phenotype and a whole-genome microarray, the purpose of the present study was to characterize, for the first time, the transcriptomic response of peripheral blood mononuclear cells (PBMCs) obtained from healthy volunteers passively exposed to a short but extreme environmental heat treatment in a sauna. The model includes young and healthy volunteers from the same Arab ethnic group, without comorbid illnesses or concurrent use of medications. Similarly, the participants were exposed to a well-defined dose of environmental heat without exposure to ultraviolet (UV) light. It was thought that a better understanding of the molecular mechanisms of human CSR to heat alone could help form the basis of improved prevention of heat-related outcomes.

## Results

### Physiological Response to Heat Stress

**Figure 1:**
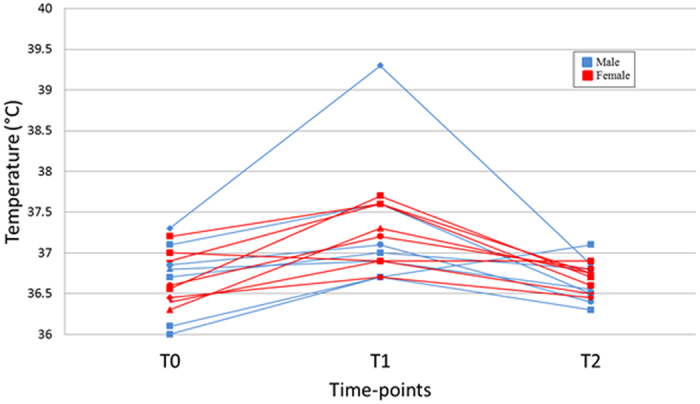
Core temperature of the study participants at baseline and after heat stress. Core temperature of 15 participants before exposure to heat stress (T0), immediately after heat stress (T1), and one hour after heat stress (T2). Heat stress was induced by passive exposure to heat in a pre-warmed sauna at temperature of 75.7 ± 0.86 °C with a humidity of 20–40% for a total of 15 minutes.

The physiological characteristics of all of the participants are presented in Table 1. Compared to females (n = 8), males (n = 7) were heavier, taller and had a higher level of fitness, as assessed by standard cardiovascular stress. The mean (SD) temperature of the sauna room was 75.7 ± 0.86 °C at onset and 75.9 ± 1.07 °C at the end of heat exposure. Eleven of the 15 participants completed their 15 minutes of exposure to heat while four female participants left the sauna after 10, 10, 13 and 14 minutes. Four female and three male participants complained of headache, dizziness and/or nausea immediately after heat stress. The core temperatures of all participants were higher at the end of the heat stress than beforehand, but all temperatures remained within the normal range, except for one male subject whose core temperature increased to 39 °C at the end of the treatment (Fig. 1). After one hour of recovery, the core temperature of all but one of the participants decreased to below the pre-treatment temperature; without reaching statistical significance. Other vital signs, such as arterial blood pressure, heart rate, and respiratory rate (see Supplementary Table 1), were not statistically significantly different between males and females either at the baseline or after heat stress; as well as before and immediately after heat stress.

**Table 1:** Physiological characteristics of the study population at baseline.

Characteristics*	Male (n = 7)	Female (n = 8)	ρ value
Age (years)	23 ± 3.4	20.7 ± 2.3	0.16
Weight (kg)	79.6 ± 20.9	59.1 ± 8.9*	0.01
Height (cm)	172 ± 6.1	161 ± 5.9*	0.008
BMI (kg/m^2^)	27 ± 6.9	25 ± 3.3	0.80
Stress Test			
*Bruce (min:Sec)	10.5 ± 2.03	8.3 ± 1.21*	0.02
METS	12.6 ± 2.04	10 ± 1.5 *	0.02
Predicted Heart Rate (%)	89.90 ± 4.07	93 ± 9	0.44

Values are expressed as the mean ± SD. Comparison was made using exact Kruskal Wallis Test. BMI, body mass index

*BRUCE protocol is a treadmill exercise stress test developed by Bruce *et al*. to evaluate cardiovascular fitness^72^. After placement of a 12-lead ECG leads to the chest, the treadmill is started at 2.74 km/hr (1.7 mph) and a slope of 10%. The slope and the speed are increased every three minutes, according to a standard protocol. The test is stopped when the subject cannot continue due to fatigue or chest pain. The Bruce score is the time taken on the test in minutes and fractions of a minute. METS, metabolic equivalent unit reflects the resting volume oxygen consumption per minute (3.5 ml/min/kg of body weight) for 1 MET equivalent. In Bruce protocol, the starting point (1.7 mph at 10% slope) represents 5 METs.

### Gene Expression Signature

**Figure 2:**
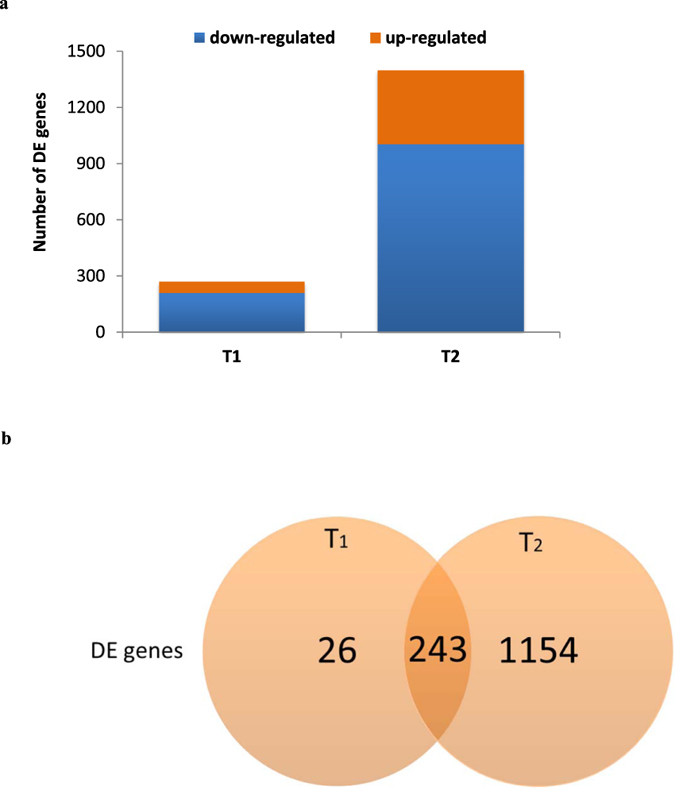
Differential gene expression after heat stress. (**a**) A bar chart depicting the number of genes that are differentially up-regulated or down-regulated immediately (T1) and one hour (T2) after heat stress relative to baseline, before exposure (T0). (**b**) Venn diagrams showing the number of DE genes immediately (T1) and one hour (T2) after heat stress. The number in the overlapping circle represents the number of genes shared by both T1 and T2.

To determine the pattern of gene expression at the three time points, a PBMC transcriptome microarray analysis, using Human Genome U133 Plus 2.0 Array chips, was performed. The analysis revealed a total of 1423 differentially expressed (DE) genes across all time points. We found 269 genes DE at T1 and 1397 genes DE at T2 of which 243 were common to both time points (Fig. 2). Exposure time and change in core temperature had no significant impact on the analysis.

There was an over-representation of downregulated genes at both T1 (n = 208; 77%) and T2 (n = 1002; 72%) (Fig. 2a). The 243 DE genes common to T1 and T2 exhibited consistent changes, up- or downregulated, at both time points (Fig. 2b). There was no statistically significant difference in the pattern of gene expression between males and females. The microarray data discussed in this study have been deposited in the NCBI Gene Expression Omnibus website (GEO; https://www.ncbi.nlm.nih.gov/geo/query/acc.cgi?token=abkrqgmonlohdch&acc=GSE90763), and are accessible through GEO series accession number GSE90763.

### Pathway and Upstream Regulator Analysis of the Heat Stress Transcriptome

#### Canonical Pathways

**Figure 3:**
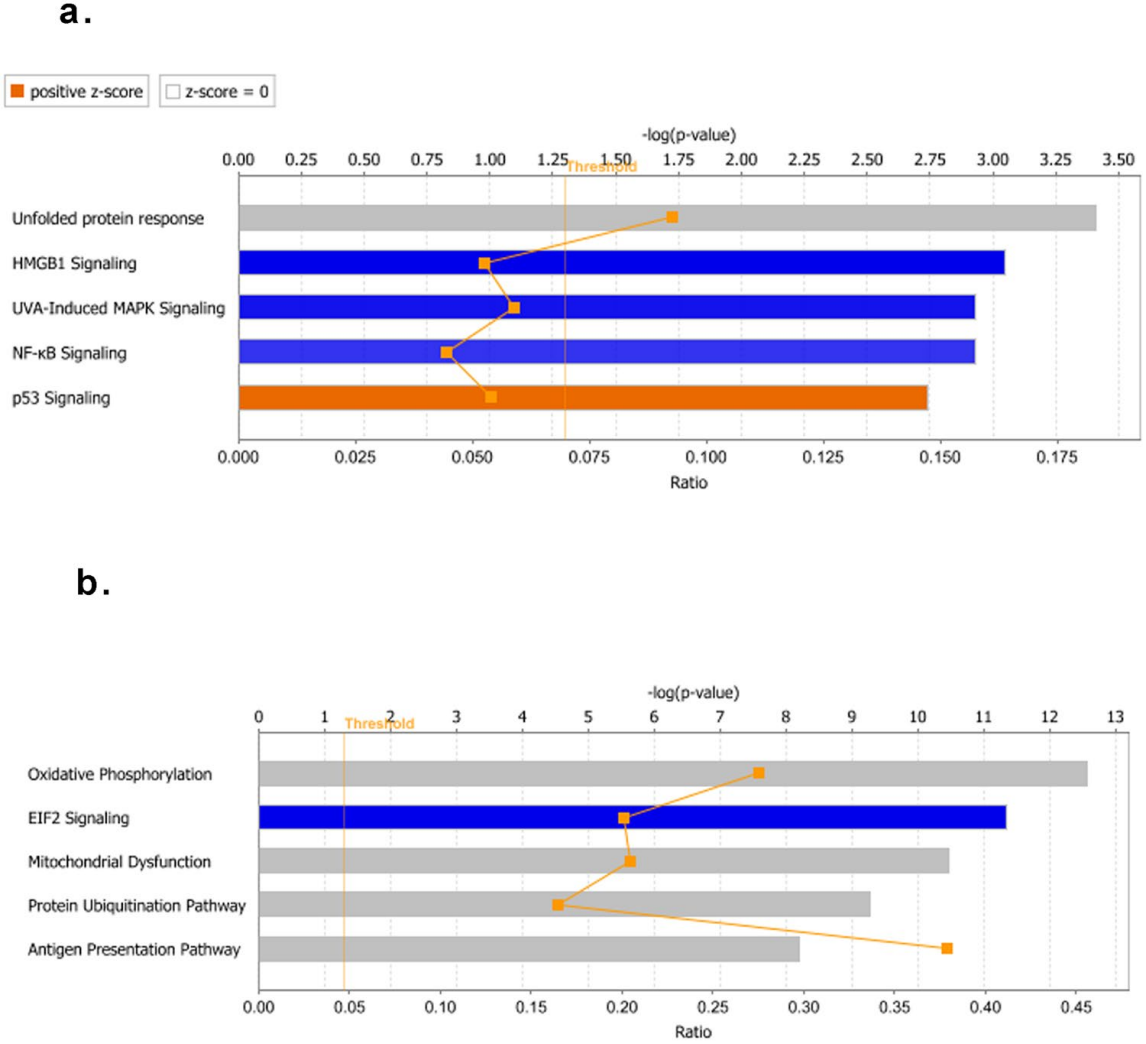
Canonical pathways identified after heat stress. Five most significant signaling and metabolic pathways identified by the IPA analysis of the differentially expressed genes, immediately after heat stress (T1) (**a**), and one hour after heat stress (T2) (**b**). The pathways are ranked by the negative log of the ρ value of the enrichment score (upper x-axis) as calculated by IPA using Fisher’s exact test, right-tailed. A Z-score ≥ 1 means that a function is significantly increased (orange) whereas a Z-score ≤ −1 indicates a significantly decreased function (blue), and an undetermined prediction in gray. The yellow straight line represents the designated significant threshold −log P value = 1.301 (ρ < 0.05). The orange curve represents the ratio values (lower x-axis) between the number of DE genes and the total number of genes in each of these curated pathways. The pathway analyses were generated through the use of QIAGEN’s Ingenuity Pathway Analysis (IPA^®^, QIAGEN Redwood City, www.qiagen.com/ingenuity).

Using Ingenuity Pathways Analysis (IPA), the DE genes were mapped to 83 and 84 significant pathways at T1, and T2, respectively (Fig. 3 and Supplementary Table 2 and 3) using a threshold ρ value < 0.05. The association of DE genes with these pathways suggests their possible mechanism of action in response to heat stress^26^.

DE genes at T1 are involved in proteostasis, bioenergetics, and cell death and survival: *HSPA1A* gene, which encodes the heat shock proteins HSP-70-1, was significantly upregulated at T1, consistent with heat shock response (Table 2)^17, 19, 20^. Other differentially expressed genes encode proteins that are known to function in a range of cellular stress -associated signaling and metabolic pathways. These include the Unfolded Protein Response (UPR), High Mobility Group Box 1 (HMGB1), Ultraviolet-A (UVA)-induced Mitogen-activated protein kinases (MAPK), nuclear factor-κB (NF-κB), and p53 signaling^27–31^ (Fig. 3a).

**Table 2:** Heat shock protein gene expression after heat stress.

Symbol	Gene Name	Exp. Fold Change T1	Exp. Fold Change T2
*HSPA1A*	Heat Shock Protein Family A (Hsp70) Member 1 A	1.342**	−1.276
*HSPB8*	Heat shock 22 kDa protein 8	1.159	1.499**
*HSPB6*	Heat shock protein, alpha-crystallin-related, B6	1.116	1.740**
*HSPB11*	Heat shock protein family B, member 11	−1.208	−1.636*
*HSP90AB1*	Heat shock protein 90 kDa alpha (cytosolic), class B member 1	−1.133	−1.671*

Values are changes (in fold log_2_ scale) in gene expression immediately after heat stress (T1), and one hour after heat stress (T2), relative to baseline, before heat exposure. Comparison was made using Generalized mixed linear model. Statistical significance *ρ < 0.05, **ρ < 0.01.

Based on the activation state of one or more DE genes in the dataset, and their causal relationships with each other derived from the curated literature, IPA predicts an activity pattern for the pathways with their end-point biological functions^26^.

**Figure 4:**
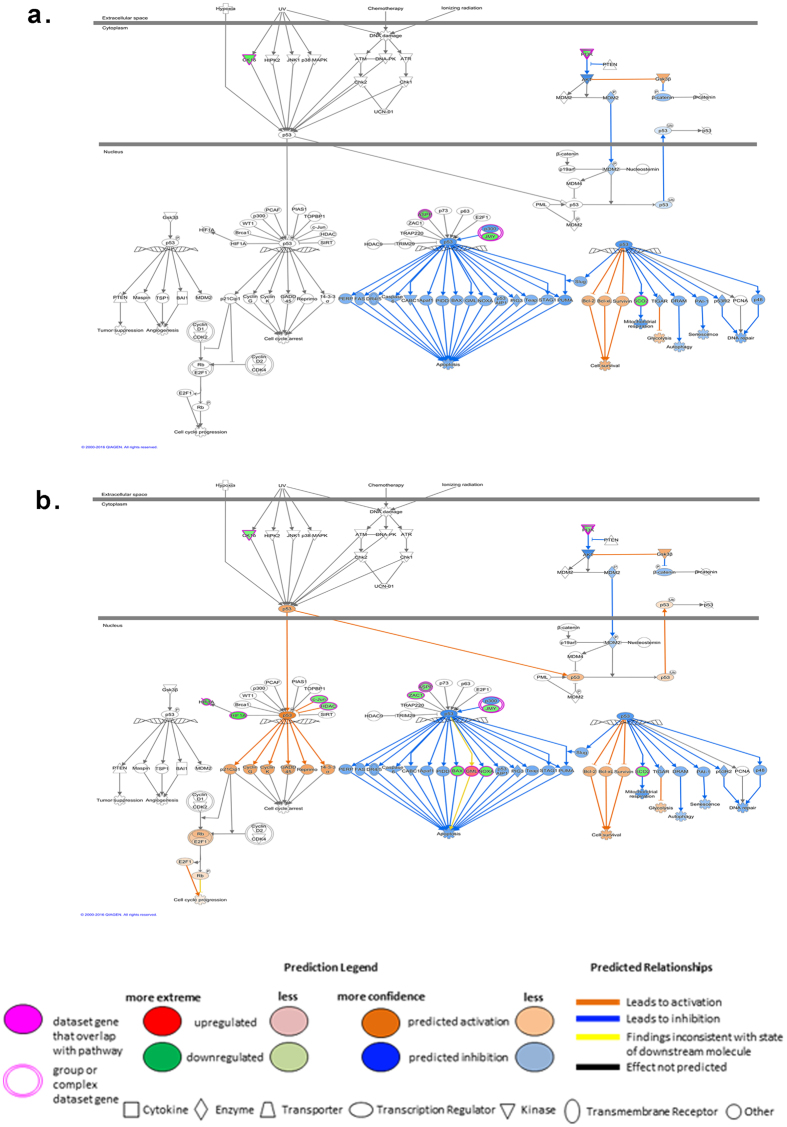
Diagram of P53 signaling pathway with overlaid molecular activity prediction after heat stress. Diagram of canonical P53 signaling pathway showing up (red) and down (green) regulated genes immediately after heat stress (T1) (**a**), and one hour after heat stress (T2) (**b**) along with predictions on biological function. Cell survival and glycolysis (colored orange) are predicted to be increased, while apoptosis, autophagy, senescence, DNA repair, and mitochondrial respiration (colored blue) are predicted to be decreased at both time points. Cell cycle progression is predicted to be increased at T2 only after heat stress (**b**). The pathway and the molecular activity prediction analyses were generated through the use of QIAGEN’s Ingenuity Pathway Analysis (IPA^®^, QIAGEN Redwood City, www.qiagen.com/ingenuity).

Accordingly, the P53 signaling pathway was predicted to be activated, with increased cell survival and glycolysis, and decrease in cell death, senescence, DNA repair, and mitochondrial respiration (Fig. 4a)^31^. The predicted activation of glycolysis with concomitant inhibition of mitochondrial respiration suggests that the energy source in heat stress relies mainly on the metabolism of glucose.

HMGB1, UVA-MAPK, and NF-κB were predicted to be inhibited with a predicted decrease in cell adhesion, inflammation and apoptotic cell death (see Supplementary Fig. 1, 2 and 3).

UPR was the most significant pathway in the early response to heat stress, although its state of activation could not be determined by IPA. However, IPA predicts that DE genes including *HSPA1A* associated with UPR lead to an increase in cell survival, suggesting that ER stress was mitigated. (see Supplementary Fig. 4)^27^.

*DE genes at T2 are consistent with alteration of protein and energy metabolism and immune response*: *HSP90AB1* and *HSPB11* genes, which encode HSP 90 alpha family class B member 1 and HSP family B (small) member 11 were significantly downregulated (Table 2). *HSPB6* and *HSPB8*, which encode the small HSPs HSP-22, and HSP alpha-crystallin-related, were significantly upregulated. The concomitant decrease in expression of *HSP90AB1* and *HSPA1A* genes suggests an attenuation of the heat shock response (Table 2).

Other differentially expressed genes were involved in bioenergetics, protein metabolism and immune response (Fig. 3b). The analysis predicted the inhibition of eukaryotic initiation factor 2 (eIF2), an important regulator of protein synthesis during stress, with a predicted increase of translation initiation, and decrease of translation elongation consistent with alteration of protein synthesis (Supplementary Fig. 5)^32^.

IPA was unable to determine the activation state of the four remaining pathways: namely oxidative phosphorylation, mitochondrial dysfunction, the protein ubiquitination, and the antigen presentation pathways. Nonetheless, there was:

- downregulation of most of the genes involved in the respiratory chain, including genes encoding the components of complexes I (NADH dehydrogenase-ubiquinone), IV (ubiquinol-cytochrome c reductase), and V (cytochrome c oxidase), with a predicted decrease in ATP production, and increase in oxidative stress (Supplementary Table 4 and Fig. 6)^33^.
- downregulation of ubiquitin conjugation and proteasome genes with predicted decrease of protein refolding, mono- and poly-ubiquitination, and antigen presentation (see Supplementary Table 5, and Fig. 7).
- downregulation of major histocompatibility (MHC) I and II complex genes, consistent with suppression of antigen presentation (see Supplementary Table 6, and Fig. 8)^34, 35^.

*Comparison analysis of canonical pathways at T1 and T2*: For a better understanding of the dynamic changes in biological processes across time-points after heat stress, we used the comparison analysis tool of IPA (see Supplementary Fig. 9).

The analysis showed that UPR was transient as the DE genes at T2 were not significantly associated with the UPR pathway. However, the prediction of increased translation initiation but with decreased translation elongation, protein refolding, mono- and poly-ubiquitination indicate a persistent proteotoxic stress^32, 34^.

The DE genes were significantly associated with the P53 pathway after heat stress, with predicted increased glycolysis and decreased mitochondrial respiration at T1 and T2, suggesting that the shift to glycolysis as a source of energy is sustained (Fig. 4b). This finding was supported by the concomitant predicted decrease in ATP synthesis by oxidative phosphorylation, and the significant association of DE genes with the mitochondrial dysfunction pathway at T2 (see Supplementary Fig. 9)^33^.

Finally, NF-κB pathways remained significantly inhibited at both time points after heat stress, consistent with a sustained inhibition of the inflammatory response^30^. In addition, at T2, there was a decreased expression of the major MHC I and II complexes genes, suggesting reduced antigen presentation (see Supplementary Fig. 9)^35^.

#### Upstream Regulators

The upstream regulator analysis is based on prior knowledge of expected effects between transcriptional regulators and their target genes stored in the Ingenuity Knowledge base^26^. Using a threshold ρ value < 0.05, the analysis revealed 80 and 256 upstream molecules that could potentially explain the expression changes observed in response to heat stress at T1 and T2, respectively (see Supplementary Tables 7 and 8).

Five upstream regulators for each time-point (10 in total) were selected based on the overlap ρ value (Table 3) and activation Z score (≥2 or ≤−2). Seven out of the 10 were related to the innate and adaptive immune response, including CD3, Interleukin (IL) 2, IL15, and CD 40 ligand^36–40^. CD3 and IL2 were sustained after heat stress at both time-points. This suggests that the regulation of the immune response is an important component of the human CSR. Other upstream regulators, including the PGR (Progesterone receptor), RICTOR (Rapamycin-insensitive companion of mammalian target of rapamycin), and MYC (Avian myelocytomatosis viral oncogene neuroblastoma derived homologue) are involved in various biological processes including cytoskeleton reorganization, actin filament regulation, metabolism, cellular growth and proliferation, as well as cell death and survival^41–43^.

**Table 3:** List of the most significant upstream regulators identified after heat stress.

Upstream Regulators	Category	Predicted Activation Z-score	ρ value of overlap
T1			
PGR	Ligand-dependent nuclear receptor	−2.702	4.53E-08
CD3	Complex	2.515	3.72E-07
IL2	Cytokine	−3.62	2.4E-06
CD28	Transmembrane receptor	2.035	3.42E-06
IL15	Cytokine	−2.779	4.43E-05
T2			
RICTOR	Other	6.611	2.79E-24
CD3	Complex	4.465	2.78E-12
MYCN	Transcription regulator	−3	3.41E-12
IL2	Cytokine	−5.281	7.49E-12
CD40LG	Cytokine	−3.889	1.66E-10

The five most significant upstream regulators identified by IPA analysis of the differentially expressed genes, immediately after exposure and one hour after heat stress. The regulators are ranked by the negative log of the ρ value of the enrichment score as calculated by IPA (www.qiagen.com/ingenuity) using Fisher’s exact test, right-tailed and activation Z-score (≥2 or ≤−2). A Z-score ≥2 predicts increased activity, whereas a Z-score ≤−2 predicts inhibited activity.

### Biological Processes, Toxicity Function and Disease

#### Molecular and Cellular Functions

**Figure 5:**
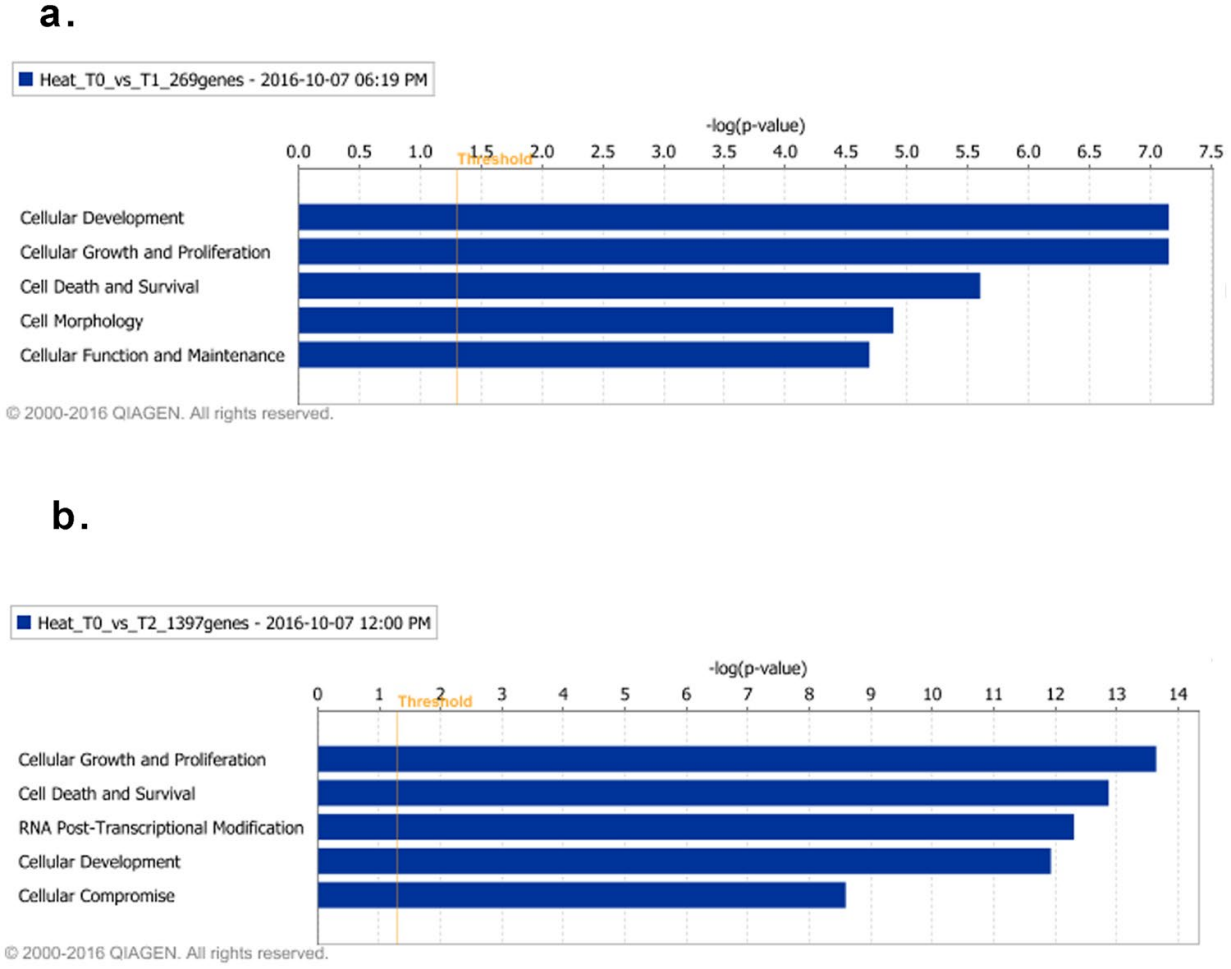
Molecular and cellular functions associated with transcriptional changes after heat stress. Biological functions associated with transcriptional changes immediately (T1) (**a**), and 1 hour after heat stress (T2) (**b**). The biological functions are ranked by the negative log of the P value of the enrichment score (upper x-axis) as calculated by IPA using Fisher’s exact test, right-tailed. The yellow straight line represents the designated significant threshold –log P value = 1.301 (ρ < 0.05). The biological function analyses were generated through the use of QIAGEN’s Ingenuity Pathway Analysis (IPA^®^, QIAGEN Redwood City, www.qiagen.com/ingenuity).

IPA analysis can predict the biological processes and functions that are likely to be affected by the gene expression changes identified after heat stress (Fig. 5). At T1, the DE genes were involved in five most significant molecular functions ranked by the overlap ρ value (Fig. 5a). These include cellular development, cellular growth and proliferation, cell death and survival, cellular morphology, and cellular function and maintenance. At T2, DE genes were involved in cellular growth and proliferation, cellular death and survival, RNA-post translational modification, cellular development and cellular compromise (Fig. 5b). Cell death and survival (Z score = −2.2 and −5.0 at T1 and T2, respectively), and cellular growth and proliferation (Z score = −2.1 and −4.5 at T1 and T2, respectively) functions were sustained after heat stress and predicted to be inhibited.

#### Toxicity Function and Diseases

**Figure 6:**
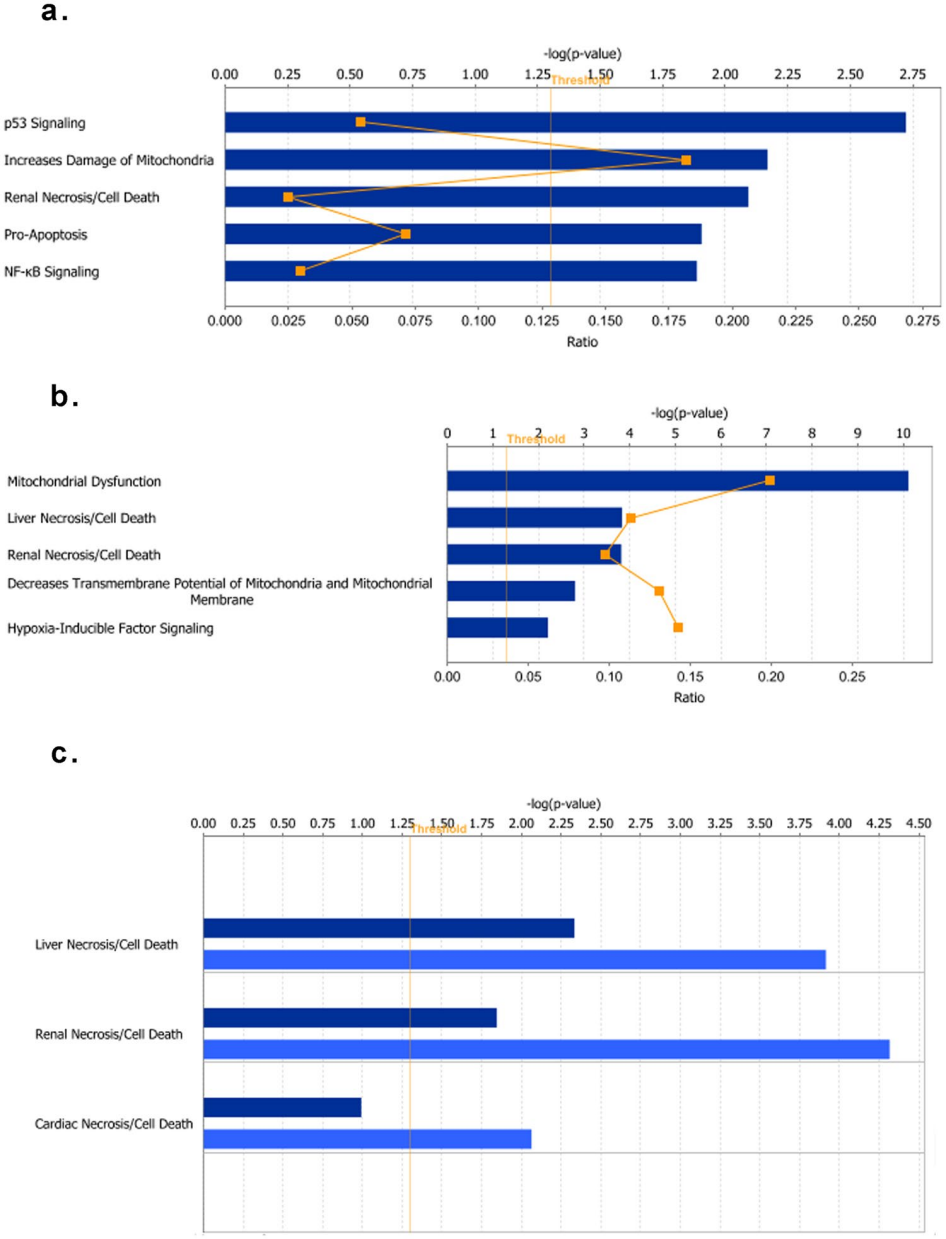
Predicted adverse signaling pathways and organ damage associated with transcriptional changes after heat stress. Predicted adverse signaling pathways identified in participants immediately (**a**) and one hour (**b**) after heat stress. The pathways are ranked by the negative log of the P value of the enrichment score (upper x-axis) as calculated by IPA using Fisher’s exact test, right-tailed. The orange curve represents the ratio values (lower x-axis) between the number of DE genes and the total number of genes in each of these curated pathways. The straight yellow line represents the designated significant threshold –log P value = 1.301 (ρ < 005). Predicted organ damage after heat stress (**c**); dark blue bars, 15 minutes; light blue, 1 hour. Organ damage are ranked by the negative log of the P value of the enrichment score (upper x-axis) as calculated by IPA using Fisher’s exact test, right-tailed. The toxic effects and disease analyses were generated through the use of QIAGEN’s Ingenuity Pathway Analysis (IPA^®^, QIAGEN Redwood City, www.qiagen.com/ingenuity).

The toxicity function of IPA was used to predict the pathological endpoints of heat stress, possibly mediated through the changes in gene expression. It also provides information about the signaling pathways mediating these cytotoxic effects. p53 signaling was predicted to be driving the toxicity function at T1 whereas mitochondrial dysfunction was most significant at T2 (Fig. 6a,b). The other significant toxicity related pathways included the cell death signaling pathway at both time points, while NFκB signaling and hypoxia-inducible factor signaling were the most significant toxicity pathways at T1 and T2 respectively (Fig. 6a,b). The most significant clinical endpoints mediated by these pathways were predicted to be in the form of hepatic, renal and cardiac toxicity (Fig. 6c).

### Validation of Microarray Data by Quantitative Real-time PCR (qPCR)

Sixteen representative genes from each time point were selected to validate the microarray data by qPCR using TaqMan probes (see Supplementary Table 9). Eleven (68.7%) out of 16 genes show the same expression pattern in real-time PCR, with comparable fold-change values to those of microarray-based expression profiling. Four genes displayed similar fold-change values, but without reaching statistical significance, and one did not.

## Discussion

The CSR to environmental heat, including changes in gene expression, plays a vital role for most organisms^10, 12–14, 17–20^. Upon stress, cells rapidly reprogram gene expression to favor stress-related genes, at the expense of growth-related genes, in order to adapt to the changing conditions; thereby averting damage and death^10, 12–14, 17–20^. We have characterized, for the first time using whole genome microarrays, the human transcriptional CSR in circulating PBMCs obtained from participants passively exposed to extreme environmental heat.

The results reveal that expression changes occur rapidly after 15 minutes of heat exposure, with no significant increase in core body temperature, and continue to amplify one hour after the end of heat stress, as the number of differentially expressed genes increases markedly. The effect of heat stress on transcription was predominantly inhibitory, with more than two-thirds of the DE genes being suppressed. At both time points, the reprogramed transcriptome comprised genes involved in stress-associated signaling and metabolic pathways, that regulate proteostasis, energy metabolism, and immune response^27, 30–34^. Gene function analysis predicted the downregulation of a range of processes relating to cell growth and proliferation as well as cell death and survival.

Taken together, the transcriptomic response pattern to environmental heat stress observed in humans is reminiscent of the stress response of model organisms, suggesting a high degree of evolutionary conservation of the CSR^12–14, 17–20^. However, the human CSR comprises alteration in mitochondrial bioenergetics, protein metabolism, and reduced immune function aimed at restoring homeostasis and yet might be potentially harmful.

What triggered the CSR in our heat stressed participants is not known. Studies using isolated cells attributed the activation of the CSR to hyperthermia and/or to heat-induced macromolecular damage, particularly misfolded proteins^19, 20^. Recent studies using multicellular organisms suggest that, similar to the thermoregulatory response, the CSR can be activated by extreme environmental heat, independently of hyperthermia and macromolecular alteration, to prevent damage and maintain homeostasis^44, 45^.

In the present study, each of the mechanisms alone and/or combined, could have contributed to the activation of the CSR. The participants were subjected to extreme environmental heat exceeding 70 °C, which led to an increase in core temperature (up to 39 °C in one participant), although, it did not reach statistical significance. Also, three of the 10 pathways identified at both time-points after heat stress by the IPA analysis, the UPR at T1, eIF2 and protein ubiquitination signaling pathways at T2, function to maintain proteostasis. These suggest that heat may have caused protein unfolding and/or aggregation^27, 32, 34^.

Genes expressed immediately after heat stress were significantly associated with the UPR pathway, suggesting that heat stress resulted in an influx of misfolded or aggregated proteins to ER that overwhelmed its protein folding capacity, resulting in ER stress, and thereby triggering the UPR^27^. The UPR was transitory as the genes expressed one hour after heat stress were not associated significantly with the UPR pathway; suggesting that alleviation of ER stress has occurred rapidly. However, there was a concomitant decrease of protein translation elongation, protein refolding and ubiquitination processes suggesting that proteostasis had not yet been restored^32, 34^.

Cells respond to stress by reprograming the transcriptome, including genes that govern the shift from rapid proliferation and growth processes to stress response and adapt metabolism to survive the stress^12–14^. Our analysis predicts that the altered gene expression observed in our participants at both time-points after heat stress results in the inhibition of both cell death and survival, and cellular growth and proliferation. This prediction is consistent with the current understanding of the CSR described in organismal models^12–14^. It also suggests several signaling pathways that could have mediated these biological processes. These include the predicted activation of p53, a well-known repressor of transcription of several growth-related genes, with concomitant prediction of downregulation of the growth and proliferation promoter pathways such as HMGB1, MAPK and NF-κB, thus, underlying the shift from cellular growth and proliferation to adaptation and survival^28–31^.

Our results suggest that the early heat stress response in human beings involves novel pathways, such as the p53 signaling pathway. The predicted activation of p53 signaling reported in the present study is consistent with previously published gene expression data obtained from human cells subjected to heat shock *in vitro* ^46–48^. Activation of p53 generally occurs in response to cellular and/or genotoxic stress and accordingly mediates graded responses^31^. These responses include regulating energy, repairing DNA, and controlling cell growth and proliferation. Cells are thereby allowed to adapt to and survive transient stress, and responses can extend to the induction of cell death or senescence^31^. Previous studies have suggested that *in vitro* heat shock induces DNA double-stranded breaks and chromatin alteration^49, 50^. Heat is also known to affect the energy balance and redox state of cells^19, 20^. Our analysis predicts that activation of the P53 pathway led to cell survival and inhibition of apoptosis, autophagy and DNA repair mechanisms. However, it remains unclear whether or not the DNA integrity was altered in our participants and there is scope for further study. Also, the analysis predicts suppression of mitochondrial respiration and activation of aerobic glycolysis controlled by P53 via the metabolic gene SCO2 (synthesis of cytochrome c oxidase), a mechanism described previously in P53 knockout mice model subjected to exertional heat stress^51^. Taken together, the findings of this study suggest that P53 may be a central regulator of the CSR to extreme heat in humans.

Mitochondria are highly dynamic organelles that provide energy in the form of ATP through oxidative phosphorylation^33, 52, 53^. Hence, mitochondrial activity is essential for cellular function and survival during stress. However, our IPA analysis identified that expression of most of the genes involved in the electron respiratory chains and in ATP production was repressed, indicative of mitochondrial dysfunction, although, the directionality of the pathway could not be determined (Fig. 3b). This finding is consistent with the previously reported reduction in ATP production during heat stress, which was attributed to the uncoupling of oxidative phosphorylation and/or a decreased number of mitochondria^20, 54–56^. Here, we extend these earlier observations by demonstrating that reduced mitochondrial bioenergetics is an adaptive mechanism regulated at the transcriptional level, possibly in part via the P53 signaling pathway^51^. Further, the analysis also suggests that upon heat stress, mononuclear cells rely on aerobic glycolysis to generate ATP, a phenomenon known as the Warburg effect, which was described in rapidly proliferating cells such as cancer or hematopoietic cells^57, 58^. The reliance on aerobic glycolysis rather than oxidative phosphorylation was recently demonstrated in stressed *Drosophila melanogaster* and attributed to UPR activating transcription factor (ATF) 4^59^. This study could not answer the question why the human CSR to heat results in a switch to a less efficient form of metabolism. One glucose molecule generates only two ATP via aerobic glycolysis and 36 ATP through oxidative phosphorylation. However, it is appealing to speculate that as mitochondrial oxidative phosphorylation is a major producer of radical oxygen species with related-tissue damage, the aim may be protective^33, 57^. The prediction that oxidative stress was increased in the present study lends support to this interpretation.

Numerous studies have documented the alteration of both innate and adaptive immune responses during stress^60–62^. This alteration has been attributed in large part to the immunomodulatory role of upregulated HSPs, particularly HSP 70 and HSP 60^60–62^. This immunomodulatory role has been explained by their extraordinary evolutionary conservation, particularly between microorganisms and human, resulting in immune cross-reactivity between exogenous HSPs and self-HSPs^62^. Thus, upregulated self-HSPs 60 and 70 act as immunogenic antigens that are presented by MHC class I to cytotoxic T -cells^62^. Another immunomodulatory role of upregulated HSPs is the downregulation of the inflammatory response demonstrated in various clinical and experimental models; although the mechanisms remain unclear^62–65^. Regulatory cytokines, such as IL4, IL10 and transforming growth factor (TGF) β, result in the inhibition of inflammation by T-cells primed to upregulated self-HSP while direct inhibition of inflammation at the transcriptional level may also play a part^63–65^.

The present study shows that the immune response is an important component of the CSR to extreme heat in human. NF-κB signaling pathway, which influences innate and adaptive immunity and inflammation, was downregulated at both time points after heat stress, suggesting a sustained inhibition of the inflammatory response at the transcriptional level^30^. Importantly, the activation of inflammation before heat stress has been shown to result in apoptotic cell death, suggesting, albeit indirectly, that modulation of the inflammatory response might have a critical role in cell survival during heat stress^10^. Likewise, at one hour after heat stress, most of the histocompatibility class I (HLA, A to F), and class II (HLA-DMA, DPA1, DPB1, DRA, and DRB1) genes were suppressed, indicating reduced antigen presentation. Further studies are needed to investigate the precise role and clinical implications of the downregulation of immune function-related genes during environmental heat exposure.

Heat-related morbidity and mortality can be observed from the third day of high temperatures and these effects accelerate markedly as a function of the intensity and duration of the heat wave^5, 6^. However, to the causative mechanism of morbidity and mortality remains poorly understood^7^. Our study was not designed to mimic the long duration of heat exposure observed during heat waves and the associated morbidity and mortality. Rather, the participants were subjected to extreme environmental heat for a short time, with a full recovery phenotype. The purpose was to examine the effect of heat alone on the stress response in a well-controlled experiment and thereby uncover the potential metabolic and signaling pathways that contribute to the clinical outcomes. The results suggest that exposure to heat alone could lead to cardiac, liver and kidney damage and/or failure, thus predicting the multiple organ injury and dysfunction observed among victims of heatstroke, and thereby validating, albeit indirectly, the pertinence of our experimental model^7^. More importantly, our analysis suggests several metabolic and signaling pathways that might underlie heat-related injury and damage. These include p53 and pro-apoptotic signaling mitochondrial dysfunction, hypoxia-inducible factor and NF-κB signaling. Further validation studies using laboratory animal models are needed to test these novel hypotheses.

There are several limitations to this study to note. First, we have used PBMCs as surrogates for whole-body, non-exertional heat stress. This approach has been validated by a number of studies of exertional heat stress, but this is the first time that PBMCs have been used in a non-exertional heat stress setting^16, 22, 24, 25^. Thus, it remains unclear whether the gene changes observed in the present study are generalizable to other cell types. For instance, hematopoietic cells are the only non-cancerous cells that exhibit the use of energy from aerobic glycolysis rather than the more efficient mitochondrial respiration, so whether this important finding in our study can be generalized requires further studies using non-hematopoietic cells^58^.

In addition, the sample population used for this study was small, particularly given the wide genetic variation between individual humans^66^. Moreover, whole-genome, microarray-based transcriptional profiling can generate a lot of background noise that might render the analysis uncertain^11, 67^. We have attempted to minimize these factors by rigorous selection of the study population in terms of age, ethnicity, fitness, and health status; together with a well-controlled heat stress procedure.

A third potential limitation of this study is the false discovery rate of the expressed genes with significant ranges from less than 1% to 16.3%; this makes it likely that some of the gene expression changes reported as significant might be false positives. Previous experimental studies have indicated that constitutive genes that undergo small-scale expression changes contribute the most to the heat shock response, rather than the strongly up-regulated and down-regulated inducible genes^11^ and so the potential inclusion of some false positives is a tradeoff for capturing some subtle but important gene changes.

Nonetheless, the present study has characterized for the first time the human gene expression response to extreme environmental temperature under passive conditions. Our findings show that the transcriptional response is prompt, dynamic and extensive, and induces a transcriptional program that could promote cell survival and restore homoeostasis. The data also suggest that the human CSR to heat stress involves mitochondrial dysfunction, altered protein synthesis, and reduced gene expression-related to immune function. How and under what circumstances these CSR-induced vulnerabilities might contribute to heat-induced adverse health outcomes requires further investigation, and this in turn might lead to improvements in the prevention and treatment of heat-related morbidity and mortality.

## Materials and Methods

### Study Participants and Heat Stress Procedure

Fifteen male and female volunteers were recruited by advertisement among Saudi Arabs studying and/or working at King Abdulaziz Medical city, Riyadh, Saudi Arabia to participate in the study. The study protocol was approved by the Institutional Review Board of King Abdullah International Medical Research Center and all methods were performed in accordance with the World Medical association declaration of Helsinki on ethical principles for medical research involving human subjects. Written informed consent was obtained, and participants were evaluated by physical screening, a cardiac stress test by exercise on a treadmill and by laboratory screening (including a complete blood count and liver, renal, coagulation and cardiac profiles). Exclusion criteria were any underlying illness, current medication or abnormal laboratory test results.

The heat stress procedure was conducted before the summer season to prevent the effect of heat adaptation. The participants were kept under the supervision of trained medical staff throughout the entire study period. Participants, in groups of three, wearing light T-shirts and shorts, were exposed to environmental heat stress in a pre-warmed sauna (Sawo, Finland) heated to the recommended temperature of 70 °C–90 °C and 20–40% humidity (thermohygrometer, Sawo, Finland) for 15 minutes, if tolerated. The participants were instructed to leave the sauna early if they experienced any discomfort, including headache, nausea, dizziness, or weakness. The participants were allowed to drink *ad libitum* throughout the experiment.

After heat exposure, the participants were allowed to cool passively at ambient temperature (23–26 °C). Vital signs, including oral temperature, blood pressure, pulse rate, respiratory rate, and oxygen saturation using a pulse oximeter, were measured at three-time points: before heat exposure (T0), at the end of heat exposure (T1), and one hour after the end of heat stress (T2). A 10 ml sample of blood drawn from the antecubital vein was collected in a sodium heparin-treated tube (BD Biosciences, USA) at each time point.

### Microarray Procedures and Data Preprocessing

#### PBMC Isolation

PBMCs were isolated using separation medium (Ficoll-Paque PLUS; GE Healthcare, Bio-Sciences AB, Sweden) according to the manufacturer’s instructions. The enriched PBMCs obtained by this procedure were stored at −80 °C until required.

#### RNA Extraction

Total RNA was isolated from the PBMCs obtained from each sample using the SV Total RNA Isolation System (Promega, USA), following the manufacturer’s instructions. The quantity and quality of the RNA was assessed using the NanoDrop ND-2000 spectrophotometer (Thermo Scientific, USA).

#### Microarray Analysis

Gene expression profiling was carried out using the Gene Chip® Human Genome U133 Plus 2.0 Array (Affymetrix Inc., USA). The labeling of the sample and microarray hybridization and washing were performed following the manufacturer’s instructions. Briefly, total RNA at a concentration of 80–200 ng/µl was transcribed into double-stranded cDNA, synthesized into cRNA and labeled with Cyanine-3-CTP using the IVT PLUS Reagent kit (Affymetrix Inc., USA). The labeled cRNAs were then hybridized onto a microarray and the arrays were scanned using a 7000 G scanner (Affymetrix Inc., USA).

#### Normalization and Statistical Significance

Raw cell files were processed using freely available updated chip definition files for HGU133Plus2 arrays based on Entrez genes (HGU133Plus2_Hs_ENTREZG, version 19), to map the single-probe ID to the correct Gene ID. We were able to map 54,645 probes to 19,702 genes. The data were normalized using Robust Multi-Array Average quantile normalization as implemented as open source in the R Bioconductor package version 3.2. To reach the final gene set, a non-label-specific maximum variance filtering technique was used. This method consists of creating for each gene, a vector of all expression at the three time points, and of computing sample variance based on its expression vector. All of the genes were ranked in descending order based on the computed variance, and the top quartile (4,925 genes) was retained for further statistical analysis.

### Statistical Analysis

All physiological and demographics data for the study cohort were summarized and reported in terms of means ± SD unless stated otherwise. All statistical comparisons between males and females were performed using exact Kruskal Wallis test.

To determine the differentially expressed genes, we used a 4,925 separate generalized linear mixed model in which gene expression values across time points were included as dependent variables and time index was included as an independent variable^68, 69^. For all of the models, we assumed underlying Beta distribution with unstructured correlation matrix to account for repeated measures over time within subject. Further, to control for the potential impact of variation in exposure time (i.e. duration spent inside the sauna) and the subject’s change in the core temperature from baseline, we have used another set of 4,925 where those variables were included as independent variables in all models. All genes with a ρ-value < 0.05 were considered for further analysis. To correct for multiplicity, we calculated False Discovery Rate (FDR) using the Benjamini–Hochberg step-up^70^. Mann-Whitney test was used to compare the genes detected by microarray-based expression profiling and by RT-qPCR. Differences were considered significant at ρ-value < 0.05. All analyses were conducted using SAS 9.3 software (SAS® 9.3, NC: SAS Institute Inc., USA).

### Pathways, Upstream and Downstream Effects Analysis

To interpret the biological functions of the genes that were significantly upregulated or downregulated in participants exposed to heat, 269 and 1397 DE genes at T1 and T2, respectively were uploaded to IPA software (www.qiagen.com/ingenuity). IPA generated a “Core analysis” comprising top canonical pathways, upstream regulators, biological and diseases function, and toxicity function for each time-point after heat stress.

IPA calculated an overlap ρ-value associated with each function or pathway, which estimates the probability that the association between our dataset of differentially expressed genes after heat stress and a given process is due to random chance^26^. A ρ -value < 0.05, calculated using the right-tailed Fisher exact test, indicates a statistically significant non-random association. IPA calculated also an activation Z-score, independent of the ρ -value of overlap. The Z-score is used to infer likely activation states of canonical pathway, upstream regulator and biological process based on comparison with a model that assigns random direction^26^. A Z-score > 1 indicates that a function is significantly increased, whereas a Z-score < −1 indicates a significantly decreased function.

### Quantitative Real-time PCR

Single-stranded cDNA was prepared from 1.5 μg of total RNA in a 50-μl reaction volume using the High- Capacity cDNA Reverse Transcription Kit (Applied Biosystems, USA), according to the manufacturer’s instructions. The 50 μl reaction was diluted 1:3 with nuclease- free water (Affymetrix Inc., USA) and then stored at −20 °C until the RT-PCR analysis was performed.

RT-PCR was performed using TaqMan gene expression Master Mix and TaqMan Gene Expression Assay from (Applied Biosystems, USA) according to the manufacturer’s instructions. TaqMan genes included *ARPC1B*, *ERP29*, *IRS2*, *OLIG1*, *TSC22D3*, *DDIT4*, *HSPA1A*, *MYO1G*, *PRDX5*, *SSBP1*, *FSTL1*, *CYP2A7*, *AREG*, *KLF9*, *RP9P* and *HSPB6*, and *ACTB* as the endogenous control. These genes were selected randomly among the differentially expressed genes from the microarray data. The analysis was carried out with Applied Biosystems 7900HT real-time polymerase chain reaction system. The mean fold changes in each gene for each sample were calculated using the 2^−ΔΔCt^ method, as previously described^71^.

## Electronic supplementary material

dataset 1

data set 2
