# Author Correction: A Model of Exposure to Extreme Environmental Heat Uncovers the Human Transcriptome to Heat Stress

**DOI:** 10.1038/s41598-026-40575-7

**Published:** 2026-03-03

**Authors:** Abderrezak Bouchama, Mohammad Azhar Aziz, Saeed Al Mahri, Musa Nur Gabere, Meshan Al Dlamy, Sameer Mohammad, Mashael Al Abbad, Mohamed Hussein

**Affiliations:** 1 King Abdullah International Medical Research Center/King Saud bin Abdulaziz University for Health Sciences, Experimental Medicine Department-MNGHA, 11426 Riyadh, Saudi Arabia; 2 King Abdullah International Medical Research Center/King Saud bin Abdulaziz University for Health Sciences, Colorectal Cancer Research Program MNGHA, 11426 Riyadh, Saudi Arabia; 3 King Abdullah International Medical Research Center/King Saud bin Abdulaziz University for Health Sciences, Biostatistics and Bioinformatics Department-MNGHA, 11426 Riyadh, Saudi Arabia; 4 King Abdullah International Medical Research Center/King Saud bin Abdulaziz University for Health Sciences, Research Trauma Project-MNGHA, 11426 Riyadh, Saudi Arabia

Correction to: *Scientific Reports* 10.1038/s41598-017-09819-5, published online 25 August 2017

This Article contains an error. The original Acknowledgements section is incomplete.

“This study was supported by King Abdullah International Medical Research Center (KAIMRC) through research grant RC10/140 awarded to AB.”

should read:

“This study was supported by King Abdullah International Medical Research Center (KAIMRC) through research grant RC10/140 awarded to AB. We thank Deemah Alwadaani and Haitham Alkadi from the Medical Genomic Department for conducting the microarray experiments.”

