# Supplementary material for: A Model of Exposure to Extreme Environmental Heat Uncovers the Human Transcriptome to Heat Stress
Source: Sci Rep. 2017 Aug 25;7:9429. doi: 10.1038/s41598-017-09819-5 (PMC5573409; doi:10.1038/s41598-017-09819-5)
Supplement: Supplementary file 1 — dataset 1 [file 41598_2017_9819_MOESM1_ESM.doc]

A model of exposure to extreme environmental heat uncovers the human transcriptome to heat stress

**Abderrezak Bouchama^1,*^, Mohammad Azhar Aziz^2^, Saeed Al Mahri^1^, Musa Nur Gabere^3^, Meshan Al Dlamy^1^, Sameer Mohammad^1^, Mashael Al Abbad^4^, Mohamed Hussein^3^**

^1^King Abdullah International Medical Research Center/King Saud bin Abdulaziz University for Health Sciences, Experimental Medicine Department-MNGHA, Riyadh, 11426, Saudi Arabia

^2^King Abdullah International Medical Research Center/King Saud bin Abdulaziz University for Health Sciences, Colorectal Cancer Research Program-MNGHA, Riyadh, 11426, Saudi Arabia;

^3^King Abdullah International Medical Research Center/King Saud bin Abdulaziz University for Health Sciences, Biostatistics and Bioinformatics Department-MNGHA, Riyadh, 11426, Saudi Arabia;

^4^King Abdullah International Medical Research Center/King Saud bin Abdulaziz University for Health Sciences, Research Trauma Project-MNGHA, Riyadh, 11426, Saudi Arabia

bouchamaab@ngha.med.sa

Keywords: heat stress, transcriptome, hyperthermia, stress response


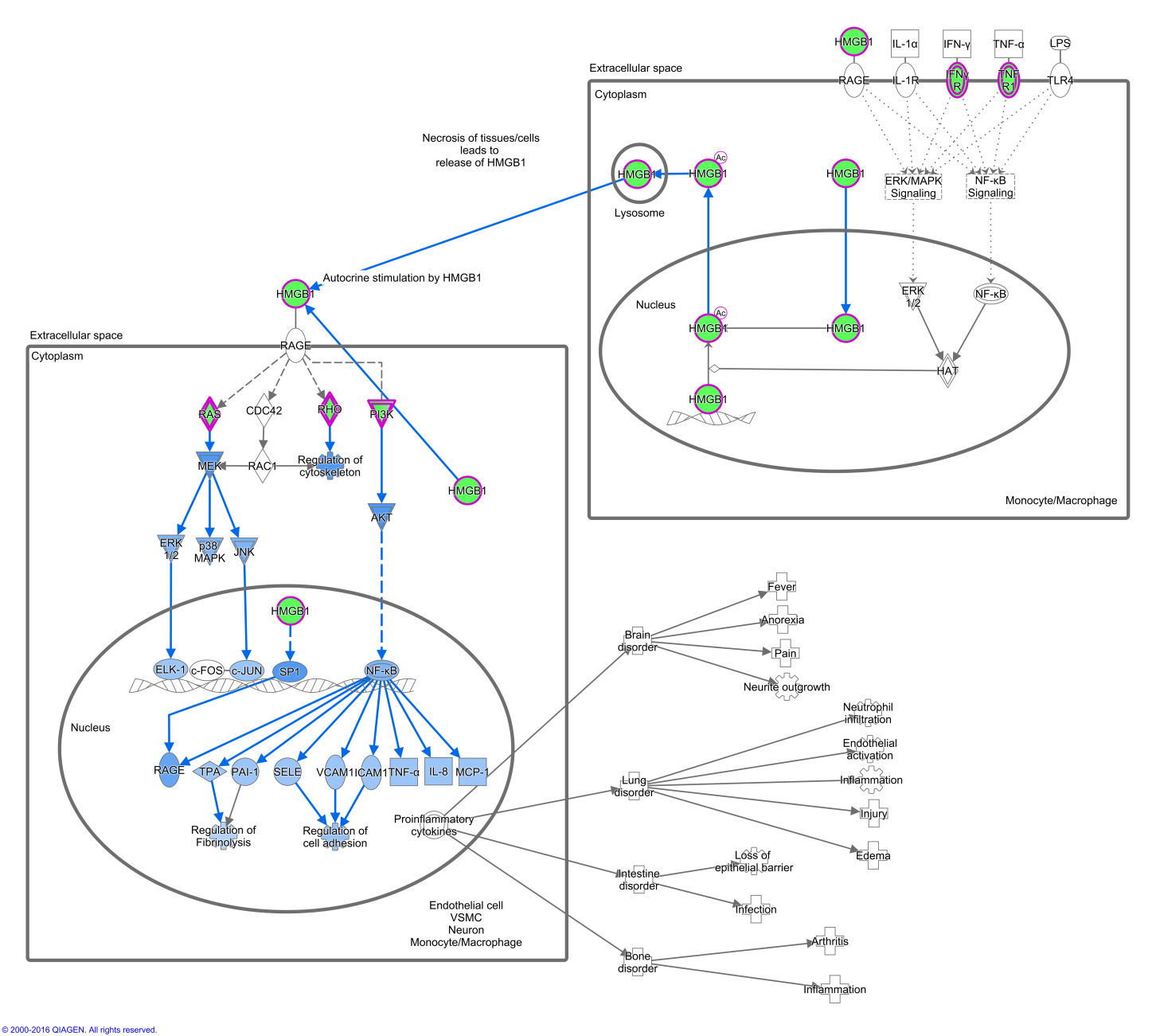


**Supplementary Fig. 1. Diagram of HMGB1 signaling pathway with overlaid molecular activity prediction after heat stress**

**
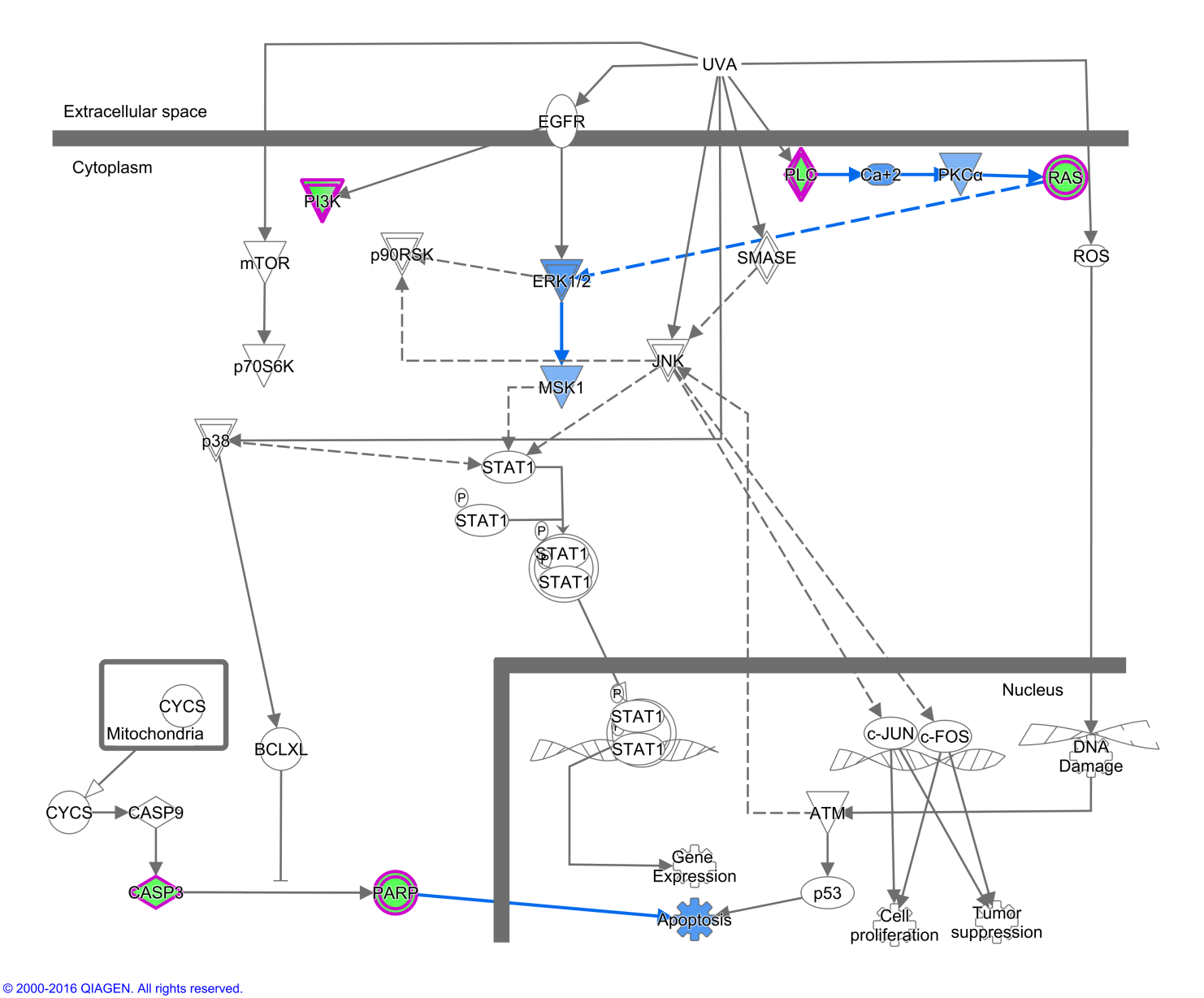
**

**Supplementary Fig. 2. Diagram of UV-induced MAPK** **signaling pathway with overlaid molecular activity prediction after heat stress**

**
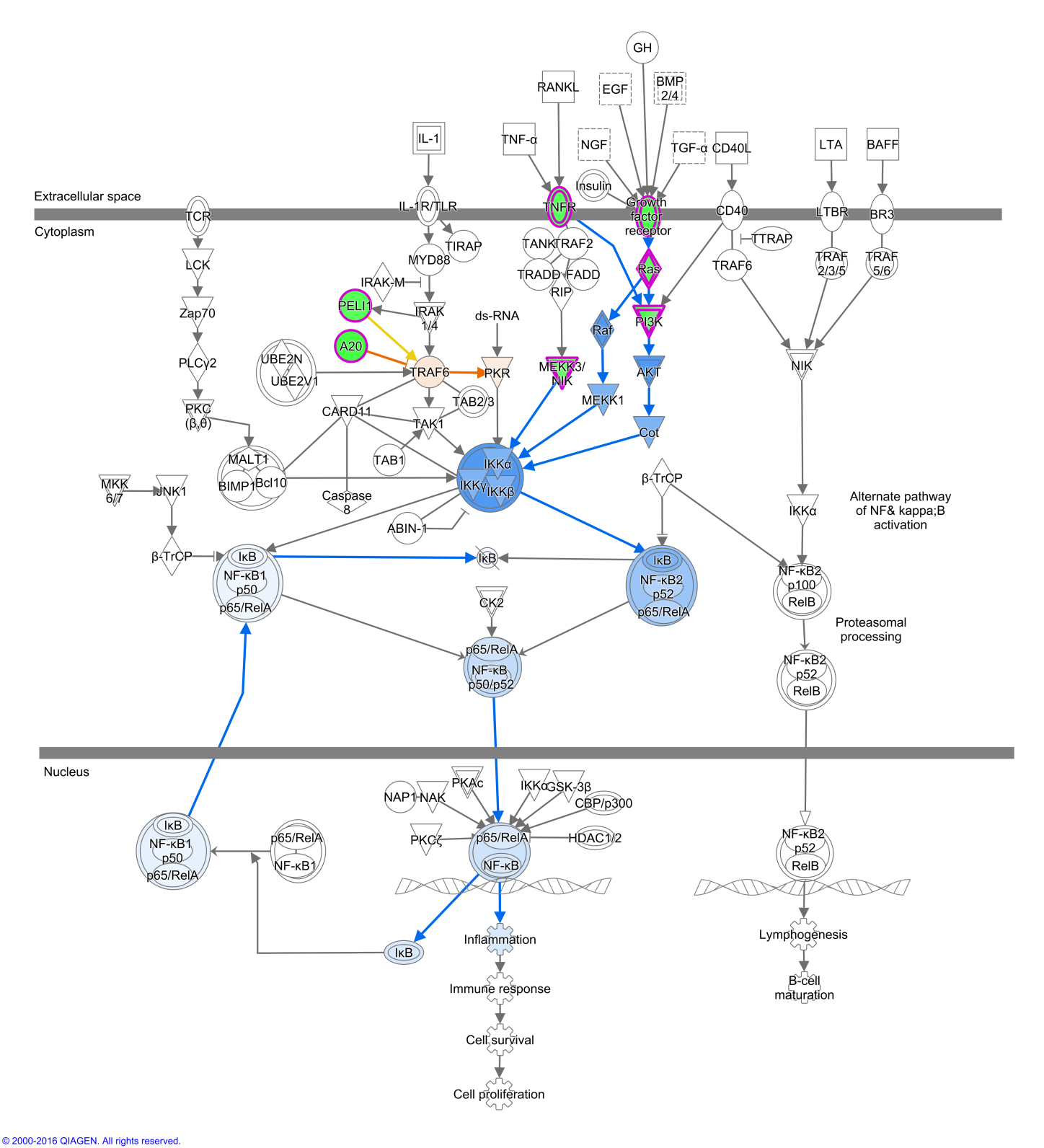
**

**Supplementary Fig. 3.**  **Diagram of NF-ĸB signaling pathway with overlaid molecular activity prediction after heat stress**


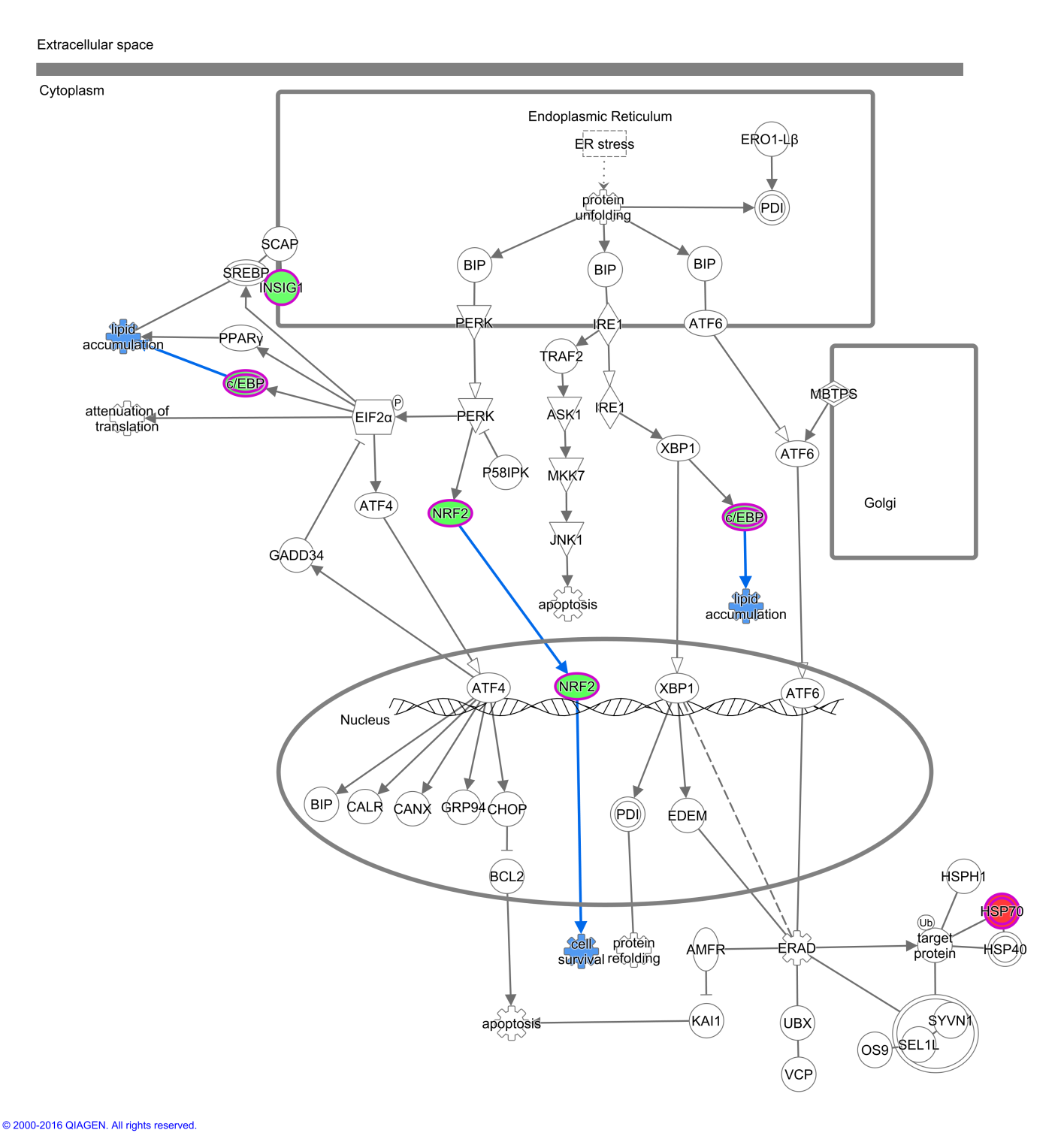


**Supplementary Fig. 4. Diagram of UPR signaling pathway with overlaid molecular activity prediction after heat stress**


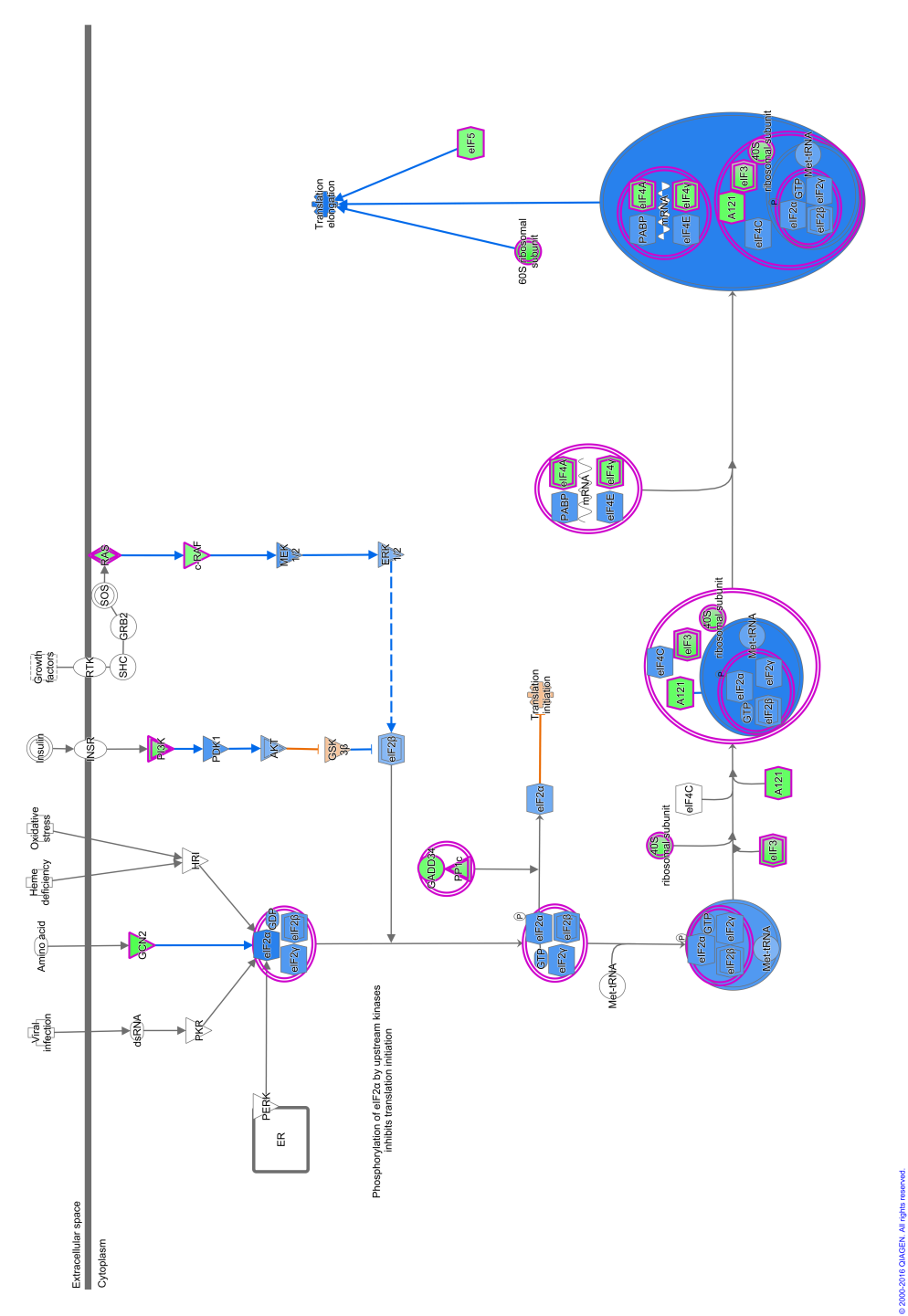


**Supplementary Fig. 5.**  **Diagram of EIF2 signaling pathway with overlaid molecular activity prediction after heat stress**


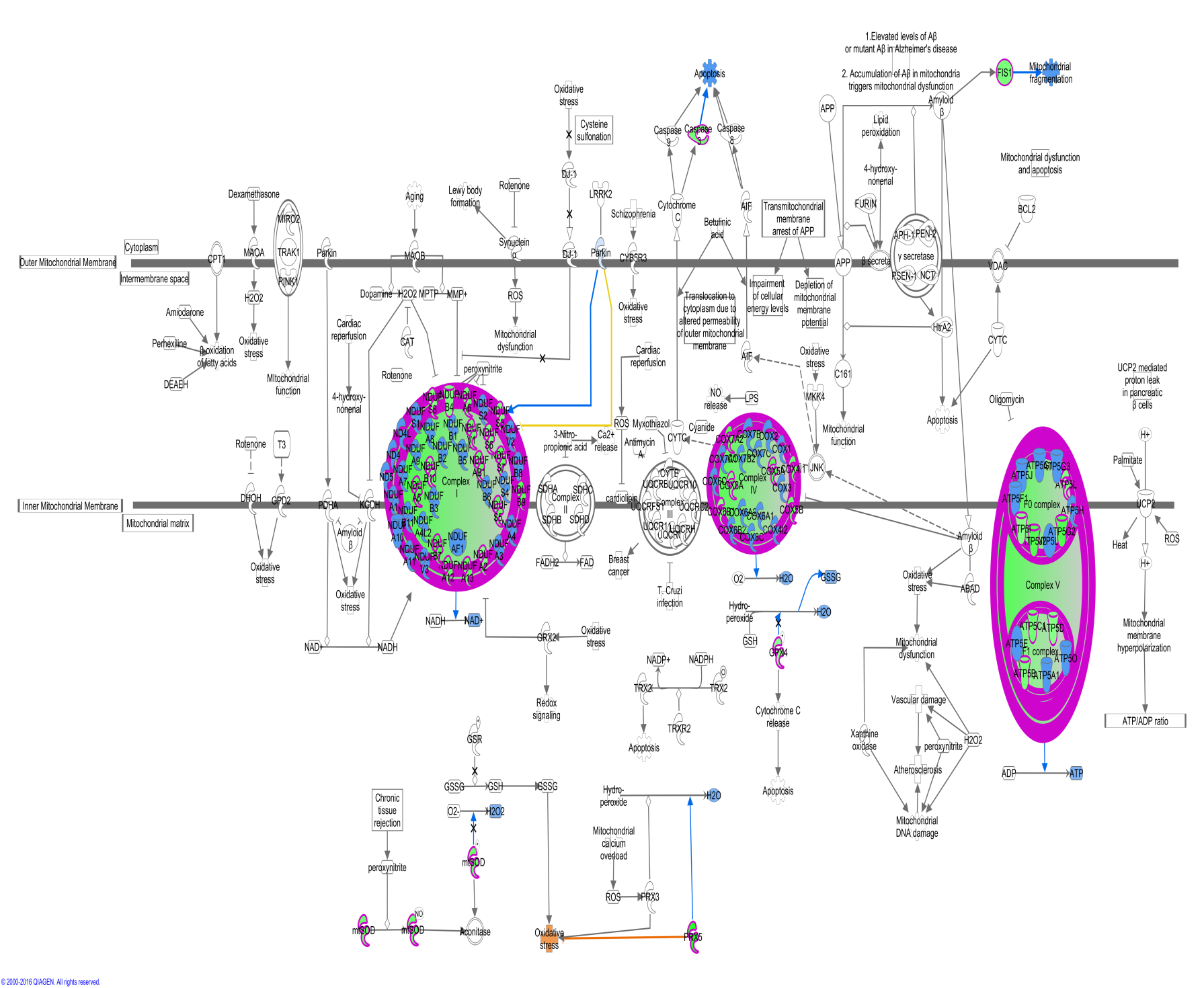


**Supplementary Fig. 6. Diagram of mitochondrial dysfunction signaling pathway with overlaid molecular activity prediction after heat stress**


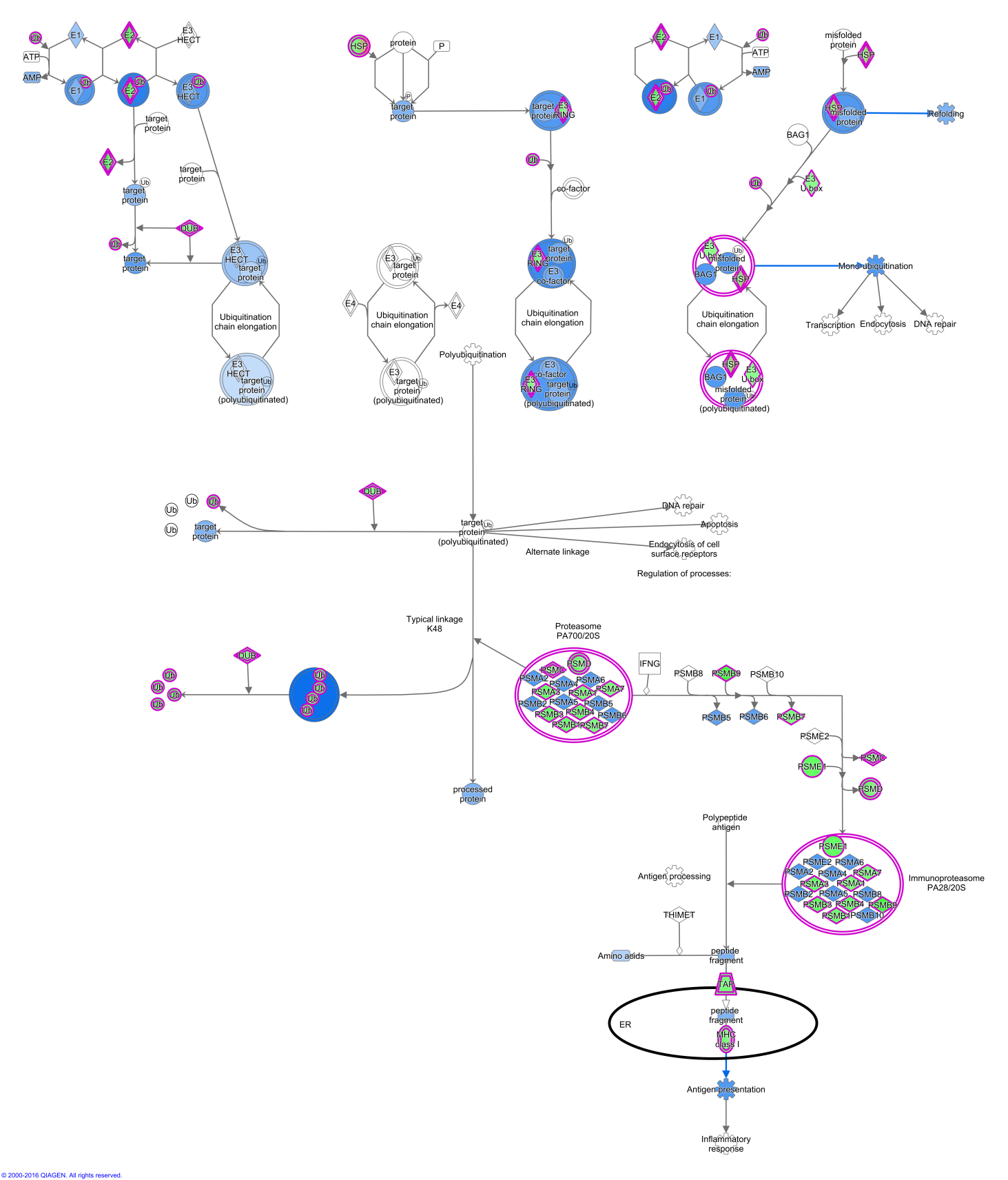


**Supplementary Fig. 7. Diagram of protein ubiquitination pathway with overlaid molecular activity prediction after heat stress**


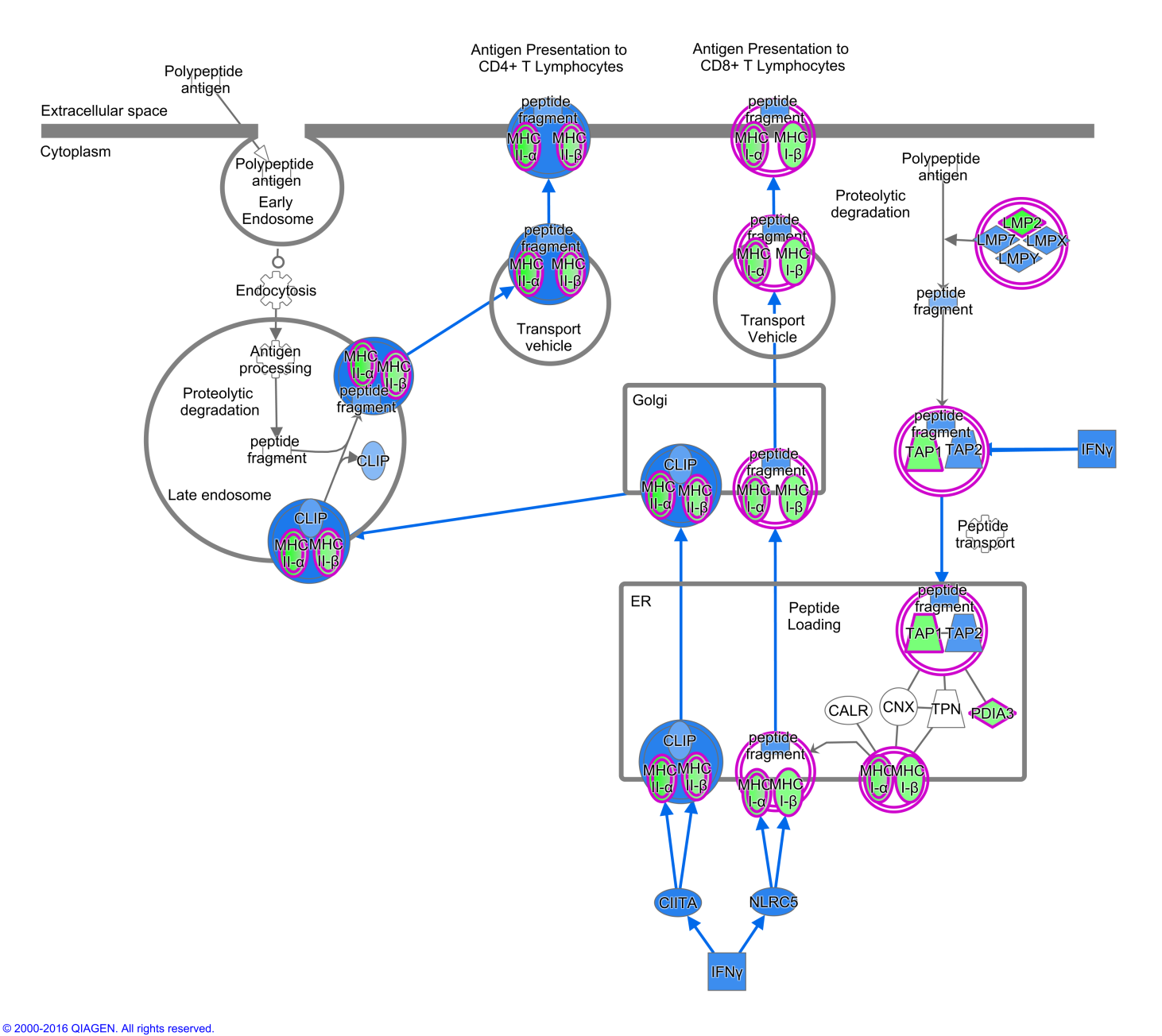


**Supplementary Fig. 8. Diagram of antigen presentation pathway with overlaid molecular activity prediction after heat stress**

**
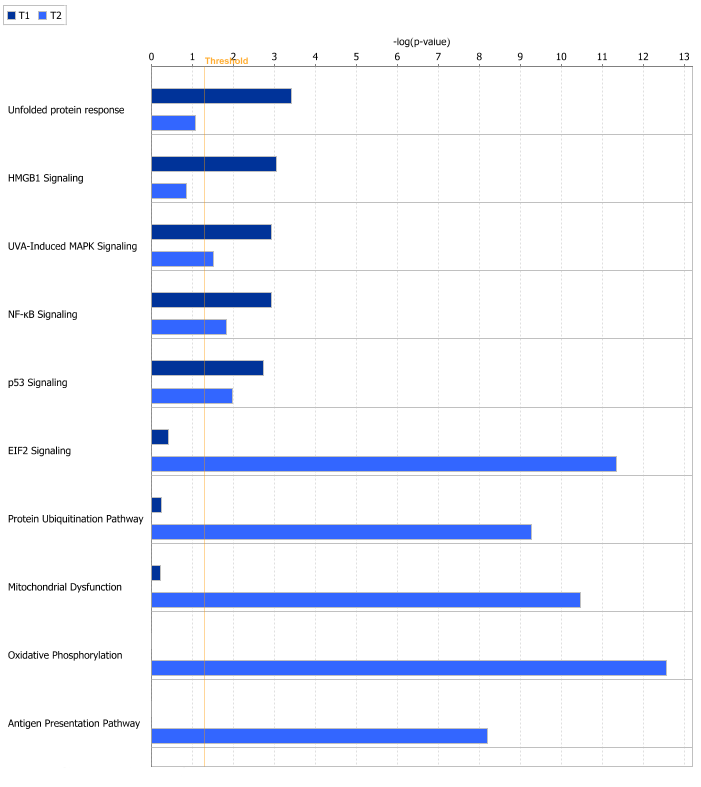
**

**Supplementary Fig. 9. Comparison analysis of canonical pathways after heat stress**

| **Characteristics** | **Male**  **(N=7)** | **Female**  **(N=8)** | **P value** |
| --- | --- | --- | --- |
| **Heat exposure**  **duration** (min) | 15 ± 0.0 | 13.6 ± 1.8 | 0.07 |
| **Temperature** (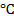) |  |  |  |
| T0 | 36.7 ± 0.5 | 36.7 ± 0.33 | 0.81 |
| T1 | 37.3 ± 0.9 | 37.2 ± 0.4 | 0.60 |
| **Heart Rate** (beats/min**)** |  |  |  |
| T0 | 109 ± 43.1 | 93 ± 9 | 0.97 |
| T1 | 100 ± 26 | 104 ± 13 | 0.95 |
| **Systolic Blood Pressure** (mmHg) | |  |  |
| T0 | 118 ± 12 | 106 ± 13.6 | 0.17 |
| T1 | 120 ± 9 | 110 ± 12 | 0.12 |
| **Diastolic Blood Pressure** (mmHg) | |  |  |
| T0 | 76 ± 8 | 70 ± 8 | 0.17 |
| T1 | 79 ± 11 | 69 ± 4 | 0.12 |
| **Respiratory Rate** (breaths/min) |  |  |  |
| T0 | 21 ± 5 | 18 ± 2 | 0.03 |
| T1 | 22 ± 6 | 18 ± 4 | 0.17 |
| **SPO_2_** (%) |  |  |  |
| T0 | 98 ± 1 | 95 ± 8 | 0.39 |
| T1 | 97 ± 1 | 94 ± 7 | 0.93 |

**Supplementary Table 1. Physiological characteristics at baseline and after heat stress**

T0 = baseline before exposure to heat stress, T1 = immediately after heat stress. Heat stress was induced by passive exposure to heat in a pre-warmed sauna at temperature of 70-90 °C with a humidity of 20-40% for a total of 15 minutes. SPO_2_ = peripheral capillary oxygen saturation. Values are expressed as the mean ± SD. Comparison was made using exact Kruskal Wallis test.

Supplementary Table 6. List of differentially expressed genes associated with Antigen Presenting Cells pathway

| Symbol | Entrez Gene Name | Exp Fold Change | Exp p-value |
| --- | --- | --- | --- |
| **B2M** | beta-2-microglobulin | -1.62 | 0.0341 |
| **HLA-A** | major histocompatibility complex, class I, A | -1.672 | 0.0234 |
| **HLA-B** | major histocompatibility complex, class I, B | -2.016 | 0.0258 |
| **HLA-C** | major histocompatibility complex, class I, C | -1.727 | 0.0307 |
| **HLA-DMA** | major histocompatibility complex, class II, DM alpha | -2.826 | 0.00947 |
| **HLA-DPA1** | major histocompatibility complex, class II, DP alpha 1 | -2.069 | 0.0292 |
| **HLA-DPB1** | major histocompatibility complex, class II, DP beta 1 | -1.672 | 0.00585 |
| **HLA-DRA** | major histocompatibility complex, class II, DR alpha | -2.54 | 0.0228 |
| **HLA-DRB1** | major histocompatibility complex, class II, DR beta 1 | -1.782 | 0.0194 |
| **HLA-E** | major histocompatibility complex, class I, E | -1.899 | 0.0168 |
| **HLA-F** | major histocompatibility complex, class I, F | -1.702 | 0.0427 |
| **PDIA3** | protein disulfide isomerase family A member 3 | -1.65 | 0.00609 |
| **PSMB9** | proteasome subunit beta 9 | -2.594 | 0.0105 |
| **TAP1** | transporter 1, ATP binding cassette subfamily B member | -1.821 | 0.0144 |

**Supplementary Table 9. Comparison between Microarray and RT-qPCR gene expression profiling**

| **Gene** | **Fold-changes (T1)** | | **Fold-changes** **(T2)** | |
| --- | --- | --- | --- | --- |
|  | Microarray | RT-PCR | Microarray | RT-PCR |
| ***AREG***** | -1.85 | -2.13 | -3.67 | -3.32 |
| ***ARPC1B**** | -1.06 | -1.29 | -3.09 | -4.63 |
| ***CYP2A7*** | 1.49 | 1.71 | 2.85 | 1.83 |
| ***DDIT4***** | -1.65 | -1.48 | -3.02 | -2.58 |
| ***ERP29***** | -1.14 | -1.30 | -3.74 | -3.68 |
| ***FSTL1****** | 1.29 | 1.13 | 1.95 | -1.32 |
| ***HSPA1A*** | 1.34 | 1.29 | -1.28 | -1.21 |
| ***HSPB6*** | 1.12 | 2.53 | 1.74 | 3.04 |
| ***IRS2**** | -1.24 | -1.32 | -1.93 | -1.14 |
| ***KLF9***** | -1.24 | -1.62 | -1.89 | -2.50 |
| ***MYO1G***** | -1.20 | -1.36 | -1.75 | -3.32 |
| ***OLIG1*** | 1.27 | 1.16 | 2.78 | -1.04 |
| ***PRDX5**** | -1.08 | -1.25 | -3.02 | -2.55 |
| ***RP9P*** | 1.30 | 1.95 | 3.70 | 1.90 |
| ***SSBP1***** | -1.19 | -1.43 | -3.17 | -2.93 |
| ***TSC22D3**** | -1.33 | -1.71 | -2.35 | -2.76 |

Quantitative real-time PCR was performed for the 16 selected genes immediately after heat stress (T1) and 1 hour after heat stress (T2). Fold-change represents the expression level of genes after heat stress relative to baseline (T0). Statistical significance was determined by Mann Whitney test. *p <0.05; **p<0.01; ***p<0.001

**Supplemental Figures**

**Supplementary Fig. 1. Diagram of HMGB1 signaling pathway with overlaid molecular activity prediction after heat stress**

Diagram of canonical HMGB1 signaling pathway showing down (green) regulated genes immediately after heat stress (T1), with regulation of fibrinolysis and cell adhesion predicted to be decreased (colored blue). Detailed legend is shown below. The pathway and the molecular activity prediction analyses were generated through the use of QIAGEN’s Ingenuity Pathway Analysis (IPA®, QIAGEN Redwood City, www.qiagen.com/ingenuity).

**
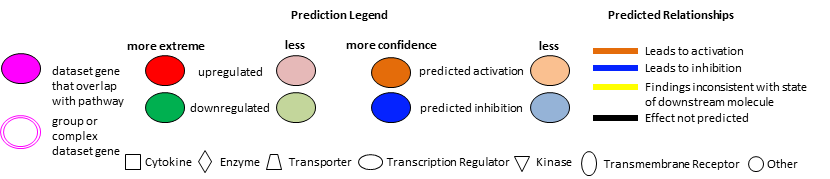
**

**Supplementary Fig. 2. Diagram of UV-induced MAPK** **signaling pathway with overlaid molecular activity prediction after heat stress**

Diagram of canonical UV-induced MAPK signaling pathway showing down (green) regulated genes immediately after heat stress (T1) , with apoptosis predicted to be decreased (colored blue). Detailed legend displayed with supplementary figure 1. The pathway and the molecular activity prediction analyses were generated through the use of QIAGEN’s Ingenuity Pathway Analysis (IPA®, QIAGEN Redwood City, www.qiagen.com/ingenuity).

**Supplementary Fig. 3.**  **Diagram of NF-ĸB signaling pathway with overlaid molecular activity prediction after heat stress**

Diagram of NF-ĸB signaling pathway showing down (green) regulated genes immediately after heat stress (T1), with inflammation predicted to be decreased (colored blue). Detailed legend displayed with supplementary figure 1. The pathway and the molecular activity prediction analyses were generated through the use of QIAGEN’s Ingenuity Pathway Analysis (IPA®, QIAGEN Redwood City, www.qiagen.com/ingenuity).

**Supplementary Fig. 4. Diagram of UPR signaling pathway with overlaid molecular activity prediction after heat stress**

Diagram of canonical UPR signaling pathway showing up (red) and down (green) regulated genes immediately after heat stress (T1), with cell survival and lipid accumulation are predicted decreased (colored blue). Detailed legend displayed with supplementary figure 1. The pathway and the molecular activity prediction analyses were generated through the use of QIAGEN’s Ingenuity Pathway Analysis (IPA®, QIAGEN Redwood City, www.qiagen.com/ingenuity).

**Supplementary Fig. 5.**  **Diagram of EIF2 signaling pathway with overlaid molecular activity prediction after heat stress**

Diagram of canonical EIF2 signaling pathway showing up down (green) regulated genes one hour after heat stress (T2), with translation initiation predicted increased (colored orange), and translation elongation predicted decreased (colored blue). Detailed legend displayed with supplementary figure 1. The pathway and the molecular activity prediction analyses were generated through the use of QIAGEN’s Ingenuity Pathway Analysis (IPA®, QIAGEN Redwood City, www.qiagen.com/ingenuity).

**Supplementary Fig. 6. Diagram of mitochondrial dysfunction signaling pathway with overlaid molecular activity prediction after heat stress**

Diagram of mitochondrial dysfunction signaling pathway showing up down (green) regulated genes one hour after heat stress (T2), with apoptosis, mitochondrial fragmentation, NAD, and ATP predicted decreased (colored blue), and oxidative stress predicted increased (colored orange). Detailed legend displayed with supplementary figure 1. The pathway and the molecular activity prediction analyses were generated through the use of QIAGEN’s Ingenuity Pathway Analysis (IPA®, QIAGEN Redwood City, www.qiagen.com/ingenuity).

**Supplementary Fig. 7. Diagram of protein ubiquitination pathway with overlaid molecular activity prediction after heat stress**

Diagram of protein ubiquitination pathway showing up down (green) regulated genes one hour after heat stress (T2), with protein refolding, mono and polyubiquitination, and antigen presentation predicted decreased (colored blue). Detailed legend displayed with supplementary figure 1. The pathway and the molecular activity prediction analyses were generated through the use of QIAGEN’s Ingenuity Pathway Analysis (IPA®, QIAGEN Redwood City, www.qiagen.com/ingenuity).

**Supplementary Fig. 8. Diagram of antigen presentation pathway with overlaid molecular activity prediction after heat stress**

Diagram of antigen presentation pathway showing up down (green) regulated MHC I and II complex genes one hour after heat stress (T2). Detailed legend displayed with supplementary figure 1. The pathway and the molecular activity prediction analyses were generated through the use of QIAGEN’s Ingenuity Pathway Analysis (IPA®, QIAGEN Redwood City, www.qiagen.com/ingenuity).

**Supplementary Fig. 9. Comparison analysis of canonical pathway after heat stress**

Ten most significant canonical pathways identified by the IPA analysis immediately after heat stress (T1) (colored dark blue) (A), and 1 hour after heat stress (T2) (colored light blue) were compared. The pathways are ranked by the negative log of the P value of the enrichment score (upper x-axis) as calculated by IPA using Fisher's exact test, right-tailed. The yellow straight line represents the designated significant threshold –log P value = 1.301 (p<0.05). The pathway and the molecular activity prediction analyses were generated through the use of QIAGEN’s Ingenuity Pathway Analysis (IPA®, QIAGEN Redwood City, www.qiagen.com/ingenuity).
